# New *Histoplasma* Diagnostic Assays Designed via Whole Genome Comparisons

**DOI:** 10.3390/jof7070544

**Published:** 2021-07-09

**Authors:** Juan E. Gallo, Isaura Torres, Oscar M. Gómez, Lavanya Rishishwar, Fredrik Vannberg, I. King Jordan, Juan G. McEwen, Oliver K. Clay

**Affiliations:** 1 Cellular and Molecular Biology Unit, Corporación para Investigaciones Biológicas (CIB), Medellín 05534, Colombia; jegallo@ces.edu.co (J.E.G.); itorres@ces.edu.co (I.T.); ogomezg@ces.edu.co (O.M.G.); mcewen@une.net.co (J.G.M.); 2 Doctoral Program in Biomedical Sciences, Universidad del Rosario, Bogotá 111221, Colombia; 3 GenomaCES, Universidad CES, Medellin 050021, Colombia; 4 School of Biological Sciences, Georgia Institute of Technology, Atlanta, GA 30332, USA; lrishishwar3@gatech.edu (L.R.); vannberg@gatech.edu (F.V.); king.jordan@biology.gatech.edu (I.K.J.); 5 Applied Bioinformatics Laboratory, Atlanta, GA 30332, USA; 6 PanAmerican Bioinformatics Institute, Cali, Valle del Cauca 760043, Colombia; 7 School of Medicine, Universidad de Antioquia, Medellín 050010, Colombia; 8 Translational Microbiology and Emerging Diseases (MICROS), School of Medicine and Health Sciences, Universidad del Rosario, Bogotá 111221, Colombia

**Keywords:** fungal diagnostics, emerging diseases, PCR assays, cross-reactions, whole genome sequences

## Abstract

Histoplasmosis is a systemic fungal disease caused by the pathogen *Histoplasma* spp. that results in significant morbidity and mortality in persons with HIV/AIDS and can also affect immunocompetent individuals. Although some PCR and antigen-detection assays have been developed, conventional diagnosis has largely relied on culture, which can take weeks. Our aim was to provide a proof of principle for rationally designing and standardizing PCR assays based on *Histoplasma*-specific genomic sequences. Via automated comparisons of aligned genome contigs/scaffolds and gene (sub)sequences, we identified protein-coding genes that are present in existing sequences of *Histoplasma* strains but not in other genera. Two of the genes, *PPK* and *CFP4*, were used for designing primer sets for conventional and real-time PCR assays. Both resulted in a 100% analytical specificity in vitro and detected 62/62 *H. capsulatum* isolates using purified DNA. We also obtained positive detections of 2/2 confirmed *H. capsulatum* clinical FFPE (formalin-fixed paraffin-embedded) samples using both primer sets. Positive control plasmid 10-fold serial dilutions confirmed the analytical sensitivity of the assays. The findings suggest that these novel primer sets should allow for detection sensitivity and reduce false positive results/cross-reactions. New assays for detecting pathogenic fungi, constructed along these lines, could be simple and affordable to implement.

## 1. Introduction

Histoplasmosis is a systemic fungal disease caused by the inhalation of conidia of the dimorphic fungus *Histoplasma capsulatum* sensu lato, with cases reported worldwide. Histoplasmosis is one of the most frequent fungal infections affecting persons living with HIV/AIDS. Histoplasmosis causes significant morbidity and mortality in HIV-infected individuals, particularly in those countries with limited access to rapid diagnostics or antiretroviral therapies, with a reported mortality up to 40% [1,2,3,4]. The initial pulmonary manifestations of histoplasmosis are often misdiagnosed as a bacterial or viral pneumonia or classified as another disease, e.g., tuberculosis, with time and effort spent looking for nonfungal infectious etiologies. Diagnosis has traditionally relied largely on conventional blood cultures, which are positive in only approximately 50% of cases and may take up to 6 weeks, thus delaying diagnosis and initiation of therapy [1]. A major limitation of the immunological test is that in the presence of an active infection, they are negative in up to 50% of immunosuppressed patients, especially those with AIDS [4]. Other assays such as enzyme immunoassays (EIA) for antigen detection can be performed in urine and serum samples, and some of them have recently become commercially available. Antigens tend to be concentrated in the urine, making *Histoplasma* antigen detection more reliable; however, false-positive results may appear due to cross-reaction with other mycoses such as blastomycosis (or those caused by other dimorphic Onygenales) as they overlap in endemicity [5]. Given the public health need to provide a reliable, rapid, and affordable diagnosis of histoplasmosis, there is a strong motivation to develop new and rapid diagnostic methods with high sensitivity and specificity, for example, by employing molecular techniques [6,7,8].

Fungal infections can be diagnosed on the basis of morphological, immunological, clinical, and histopathological information. Imaging can also be suggestive of fungal infections, such as X ray or CT scans. These images could show patchy pneumonitis that eventually calcifies and could form hilar lymphadenopathy. Advanced stages of histoplasmosis could develop a mass lesion that resembles a fibroma (histoplasmoma), although it would be difficult to differentiate histoplasmosis from other respiratory mycoses [1,4]. Of these procedures, histopathology can provide important diagnostic information in a relatively short period of time, but identification is typically only tentative unless complemented by specialized techniques such as immunofluorescence, or when the etiological agent has distinct unique structures. Gomori methenamine or Grocott staining is useful for *H. capsulatum* yeast identification, although via these or other methods, it is easily confused with other yeasts such as *Candida* spp., *Pneumocystes jirovecii*, *Cryptococcus neoformans*, or other infectious agents such as *Leishmania* spp. and *Toxoplasma gondii* [4,9]. The observation in any clinical sample of the small intracellular yeast is suggestive of histoplasmosis. However, some small cells such as poorly encapsulated *Cryptococcus* spp., *Candida glabrata*, *Penicillium marneffei, Pneumocystis jirovecii, Toxoplasma gondii*, and *Leishmania donovani* can be confused, leading to misdiagnosis, in particular, when histoplasmosis is caused by *H. duboisii*, which produces larger blastoconidia with a thicker cell wall than *H. capsulatum* [1,5].

DNA-based diagnosis has not yet been established as a routine clinical diagnostic tool for histoplasmosis, but is used in some reference laboratories [1,10]. One molecular assay used for the detection of *Histoplasma* spp. is a nested PCR assay based on a gene coding for a 100 kDa protein that is considered specific to *H. capsulatum* [11,12,13]. A 100 kDa PCR assay has also been applied to environmental detection of the fungus in composted organic fertilizers, soil samples from caves, and bird excreta.

A new line of interest has arisen as a result of the increased reliability of available fungal genome sequences during the last decade. Reference sequences that were utilized when some of the currently available assays were designed and validated have since been updated, sometimes with dramatic quality improvements in sequence and annotation accuracy [14]. With the recent availability of finished or draft genome assemblies, or even from unassembled Illumina raw sequence reads, new target regions can be identified for developing more accurate molecular diagnostic assays. The regions of interest for assay design should ideally fulfill two conditions: their sequences should be specific to the fungus of interest (i.e., not present as a highly similar sequence in other organisms), and should also be conserved in all strains of the fungus that might be present in clinical contexts. 

Properly designed and clinically validated assays should provide the laboratory technician and clinician with a definitive diagnosis of the fungal pathogen via a PCR assay that is easy to implement. Low-income countries can be affected in the diagnosis of fungal pathogens as a result of inadequate infrastructure and high costs of importation, high costs of healthcare, and/or limited budgets of local healthcare facilities/research centers [15], and the application of molecular assays in clinical settings should, if possible, not be limited to highly specialized reference laboratories. Although molecular biology equipment is costly, a local research center or small clinic may be able to easily acquire a thermal cycler for conventional PCR, or if the budget permits, the equipment needed for real-time PCR. 

With possible economic or time constraints in mind, we aimed to design a diagnostic method using either conventional PCR or real-time PCR that does not require sequencing, and we reasoned that an easily deployable and affordable assay would be beneficial for the public health sector. Considering that the development of molecular assay methods for the diagnosis of fungal infections has sometimes been guided by anecdotal reports of promising loci rather than by rational principles, the present work explored a new route. We describe here a molecular diagnostic approach that should be capable of a high level of analytical specificity and sensitivity, and a high expected clinical specificity and sensitivity, in this case, for detecting the endemic genus *Histoplasma* in tissues where the fungus can be expected to be present. The novel strategy for rationally designing PCR assays should be particularly useful where the target fungus is closely related to distinct fungi, e.g., presenting different clinical manifestations or with different recommended therapies, and, in this sense, the choice of *Histoplasma* is an especially stringent test of the principle. Indeed, the Ajellomycetaceae family, which includes *Histoplasma*, also includes the causal agents of paracoccidioidomycosis, blastomycosis, adiaspiromycosis/emmonsiosis, and the emerging disease emergomycosis, fungi that could therefore cross-react if the genomic target region of the assay is not carefully chosen with this possibility in mind. The strategy can be applied where accurate whole genome sequences of multiple strains of the target species and its close relatives are available and can be aligned.

## 2. Materials and Methods

### 2.1. Finding of Regions Unique to H. capsulatum

The methodology utilized to find genome regions that are unique to *H. capsulatum* was based on bioinformatic strategies explored by our group in the context of the closely related pathogenic fungal genera of the Ajellomycetaceae family (Supplemental Table S1). We initially used whole genome alignment programs such as NUCmer and PROmer from the MUMMER package [16] and BLAST [17], in order to identify regions that are unique to *H. capsulatum*, and absent or only poorly conserved in *Paracoccidioides* spp. [18], *Blastomyces dermatitidis*, *Emmonsia crescens*, *E. parva/B. parvum*, as well as in outgroups within the Onygenales order, nonfungal pathogens, and humans. For *H. capsulatum*, all publicly available genome sequences, including sequences from diverse strains within species, were included in the comparative genome analyses. The fungal species listed above must be considered for possible cross-reaction because of their phylogenetic proximity to *Histoplasma* spp. and/or the likelihood that they might be present in similar clinical settings. 

Three approaches were considered in order to search for *Histoplasma*-specific regions within current assemblies.

In the first approach, contigs were compared. All contigs/scaffolds of the reference assembly of *H. capsulatum* were aligned to contigs/scaffolds of the other fungal species, particularly those of the closely related pathogen *Paracoccidioides* spp., where we could vouch for high sequence quality [14], followed by a second-pass verification for analytical sensitivity by aligning all available strain sequences of *H. capsulatum* to ensure intraspecies inclusion, and then a final filtering step to ensure that they were not similar to known sequences of other pathogens or to human genomic DNA, via BLAST versus the nonredundant (nr) NCBI database.

The second approach identified genes that were unique to *Histoplasma*. Protein-coding genes confirmed or predicted by gene annotations were used to search for genes that are present in *H. capsulatum* but not in other fungal species; in these searches, we also used orthologous gene clusters obtained using OrthoMCL [19], and a final step used a BLAST search versus nonredundant databases to verify that the gene or genes selected are not present in any known sequences of other organisms. 

In the third approach, chromosomal segments of a specified length were sought that were unique to *Histoplasma*. We used a pairwise alignment algorithm based on a sliding window BLAST (with window sizes 500 and 250 bp) in order to search for sequences in *H. capsulatum* assemblies that are conserved within and only within *H. capsulatum* strains, which generates a cluster of genomic regions having a specified similarity range. 

For all three strategies, *H. capsulatum* sequence segments that meet the criteria of no similarity in the non-*Histoplasma* assemblies, and that also meet the criteria of being conserved in the strains of *H. capsulatum*, are then considered to be potential candidates for primer design.

### 2.2. Strains

Genomic DNA from fungal strain cultures listed in Table 1 were obtained from several fungal pathogen DNA collections maintained at the Corporación para Investigaciones Biológicas (CIB, Medellín, Colombia) or the Centers for Disease Control and Prevention (CDC, Atlanta, GA). Genomic DNA for microbial strains used in analytical specificity tests were also obtained from these collections and are listed in Table 1. The relative concentrations of the genomic DNA were determined with a NanoDrop ND1000 apparatus (Thermo Scientific, Wilmington, NC, USA).

### 2.3. Obtaining DNA from Clinical Samples

Formalin-fixed paraffin-embedded (FFPE) skin tissues from two patients from Clínica CES, Medellin, Colombia and previously diagnosed with disseminated histoplasmosis were analyzed. Samples were identified as 173196 and 5177. Both samples were recovered from skin biopsies and both patients had been previously classified as being HIV-positive and with AIDS. DNA extraction and purification were performed with the QIAamp DNA FFPE tissue kit (Qiagen; Valencia, CA, USA), with the following minor modifications of the manufacturer’s protocol: (i) DNA was purified from ten sections per sample, (ii) the samples were incubated with 20 μL of Proteinase K (20 mg/mL, Qiagen) overnight (almost 17 h) at 37 °C instead of 1 h at 65 °C, (iii) the DNA purification was performed with phenol:chloroform:isoamyl alcohol [20] and precipitation with sodium acetate and isopropanol, and (iv) the pellet was re-suspended in 50 μL of TE 1X.

### 2.4. Primer Design

The primers were designed using the *Histoplasma*-unique regions selected via the bioinformatic analysis and were subsequently analyzed using OligoAnalyzer 3.1, adhering to quality control guidelines provided by Integrated DNA Technologies, Inc. (IDT). Some quality control aspects that were checked include: primers must be between 20 and 23 nucleotides in length, the ideal GC content of primers should be between 40 and 60%, melting temperatures (T_m_) of primers should be between 42 and 65 °C, primers in a pair should have T_m_’s within 2 °C of each other, and secondary structures (i.e., hairpins) within primers and potential dimerization between the primers should be avoided.

The primers designed were subjected to a BLAST search against the GenBank sequence database, in order to avoid cross-homology with other microorganisms or the human genome. The primers that were selected are listed in Table 2. These primers were designed for use in the conventional and real-time PCR assays.

### 2.5. Conventional PCR Assay

The genomic regions found to be unique for *H. capsulatum* by computational prediction were experimentally assessed by conventional PCR. Thermocycler conditions were standardized via a temperature gradient of 54 °C–60 °C using a T100 Bio-Rad Thermal Cycler. The amplification products were analyzed on agarose gel and visualized with ethidium bromide under UV light. The PCR conditions selected were as follows: an initial step of 95 °C for 10 min, followed by 40 cycles of 95 °C for 30 s, 60 °C for 30 s, and 72 °C for 1 min.

### 2.6. Real-Time PCR Assay

Real-time PCR (qPCR) was performed using SYBR Green Real-Time PCR Master Mix, according to the manufacturer’s instructions (Thermo Fisher Scientific Inc.: Waltham, MA, USA) and using conditions standardized via conventional PCR. The CFX96 Real-Time PCR Detection System (Bio-Rad, Headquarters Hercules, CA, USA) was used to carry out the amplification. PCR reactions were performed in a 20 μL final volume containing qPCR master mix 2x. Each experiment was carried out in triplicate. The real-time PCR conditions were as follows: an initial step of 95 °C for 10 min, followed by 45 cycles of 95 °C for 30 s, 60 °C for 30 s, and 70 °C for 1 min, with a melting curve at 60 °C to 95 °C in increments of 0.5 °C each 0.05 s.

### 2.7. Determining Analytical Specificity of Primers In Vitro

The analytical specificity of the primer sets was evaluated by conventional PCR and corresponding real-time PCR using purified DNA from different isolates of *H. capsulatum*, as well as from collections of other related fungal pathogens and *Mycobacterium tuberculosis* maintained by the Corporación para Investigaciones Biológicas (CIB) and the Centers for Disease Control and Prevention (CDC, Atlanta, GA, USA). The isolates were tested at a concentration of 1 ng/μL. For analytical sensitivity tests, all strains of *H. capsulatum* were tested for amplification with our chosen primers. A total of 62 *H. capsulatum* isolates were used, including isolates from North America (*H. capsulatum* CDC/Thon and *H. capsulatum* G217B), Central and South America (*H. capsulatum* CIB 1980, *H. capsulatum* G184B, and *H. capsulatum* CDC 3670/CDC2787), and Africa (*H. duboisii* CDC5822/CDC5823). Isolates of other pathogens used for determining analytical specificity are listed in Table 1.

### 2.8. Generation of Positive-Control Plasmids

Positive-control plasmids were constructed for *H. capsulatum* using the primers designed from species-specific regions or genes, as described in Table 2. The amplified targets were cloned into the pCR 2.1 vector using the pCR 2.1 TOPO TA cloning kit (Invitrogen Corporation: Carlsbad, CA, USA) according to the manufacturer’s instructions. The plasmid construct was then purified using the PureLink^®^ Quick Plasmid Miniprep Kit (Thermo Fisher Scientific Inc.: Waltham, MA, USA). The ligation reactions were transformed in TOP10 chemically competent cells. Colonies were selected in LB plates containing 50 μg/mL of kanamycin (two for each transformation [21]).

### 2.9. Determining Diagnostic Sensitivity

The diagnostic specificity was evaluated by conventional PCR and corresponding real-time PCR via two routes: (i) by testing a dilution series of *H. capsulatum* control plasmids, where a 10-fold serial dilution of the plasmid was performed in TE buffer (10^5^ copies/μL serially diluted to 10 copies/μL) and was used to construct the standard curve for the limit of detection (LOD), and cycle threshold (CT) values for each dilution series were determined in triplicates in 3 different experiments consisting of 3 different tubes corresponding to the specific dilution of the curve on 3 different days; (ii) by testing DNA obtained from two FFPE skin tissues from two patients previously diagnosed with disseminated histoplasmosis and AIDS, provided by Clínica CES Medellín, Colombia.

## 3. Results

### 3.1. In Silico Assay Design

We implemented bioinformatic approaches to search for regions within the genome of *H. capsulatum* that are unique to this species (a criterion needed for high specificity), as well as likely to be present in all strains of these species (a criterion needed for high sensitivity), and that could therefore be used for the identification of *H. capsulatum* via PCR assays. These allowed us to find several regions that were optimal for primer design.

First, we searched for entire *H. capsulatum* scaffolds that were not similar to genomic regions of other species. In *H. capsulatum*, we found scaffolds, i.e., large contiguous regions (HcG186AR contig 2.315/supercontig 2.50, length = 22,664 nt; HcG217B contig 171, length = 13,544 nt; HcH143 contig 2.108 in supercontig 2.1, length = 8250 nt; HcH88 scaffold 455, length = 1881 nt; https://www.broadinstitute.org/fungal-genome-initiative/histoplasma-genome-project) that met the criteria of not aligning to other closely related fungal species and of being present in all of the *H. capsulatum* strains queried. It was, however, not possible to design primers within these regions, because of the low yield of perfect alignment stretches longer than 20 base pairs that were conserved among the *H. capsulatum* strain sequences used. This low yield may be partly due to sequencing and/or assembly errors, e.g., single nucleotide errors (SNEs) masquerading as true SNPs, which may reflect the limited base-level accuracy of the assemblies currently available for *H. capsulatum*.

We next used, as a second approach, a search for entire protein-coding genes that are unique to *H. capsulatum*. This second approach allowed us to design two promising PCR primer pairs for the detection of the species, in regions within the two genes coding for culture filtrate protein 4 (*CFP4*; HCAG_06604; [22]) and a predicted protein kinase (*PPK*; HCBG_02218). The decision to focus on these two regions was based on the genes’ annotations having a gene function associated to them, as many of the other genes had no name or specified function. The *CFP4* gene had been previously described as having potential as a diagnostic exoantigen [22]. The genes were found using OrthoMCL matrix results. The matrix produced was queried for genes that did not have any likely orthologs in other screened species. We used strict filtering for the selection of the genes: only genes that had no similarity to potentially orthologous genes in the species queried were considered for further analysis. Genes that met this criterion were then queried by BLASTN (against the NCBI nr database in order to check whether the genes were unique to *H. capsulatum*). 

The implementation of our second approach yielded many regions with sufficient length for primer design. Although, in theory, any of the regions discovered could have been used, we chose the two genomic regions from nontrivially annotated genes, as they seemed most likely to play a biological role and to be robustly genus-specific due to a functional differentiator contrasting with closely related taxa. 

The primer details and PCR conditions used are listed in Table 2. For real-time PCR contexts, we did not consider designs also involving a sequence-specific probe for a region between the two primers, as our aim was to keep costs low without sacrificing specificity. Instead, we focused on the use of DNA intercalating dyes such as SYBR Green. PCR conditions were the same for conventional and real-time PCR.

The third approach we used, employing a sliding window, served as a cross-check to confirm the uniqueness of regions reported by the first two search strategies. The use of the algorithm also permitted the discovery of other unique genomic regions that were not found using the first two strategies, although we did not investigate them further in this study.

### 3.2. Assessment of Analytical Specificity and Sensitivity of Primers Using Fungal Genomic DNA and a Positive Plasmid Control

We tested our assay designs with 62 *H. capsulatum* strains from the fungal DNA collections of the CDC and the CIB. The outcomes were positive for all of the 62 strains tested, i.e., the primer pairs had a 100% analytical specificity in vitro. The amplification was 100% specific to *H. capsulatum* as no amplification was observed in any of the other 25 species we included in the test (Table 1), for both primers.

The PPK primer pair resulted in PCR products having homogeneous amplicon sizes of approximately 400 bp in all the tested DNAs. The CFP4 assay resulted in PCR product amplicons of approximately 800 bp, although a few strains had amplicons reaching up to 1000 bp when testing in positive control plasmids. Considering that smaller fragment sizes (i.e., lower molecular weights) of the target DNA might often be present in preparations from clinical samples, we recommend primarily the PPK primer pair instead of the CFP4 primer pair.

**Figure 1:**
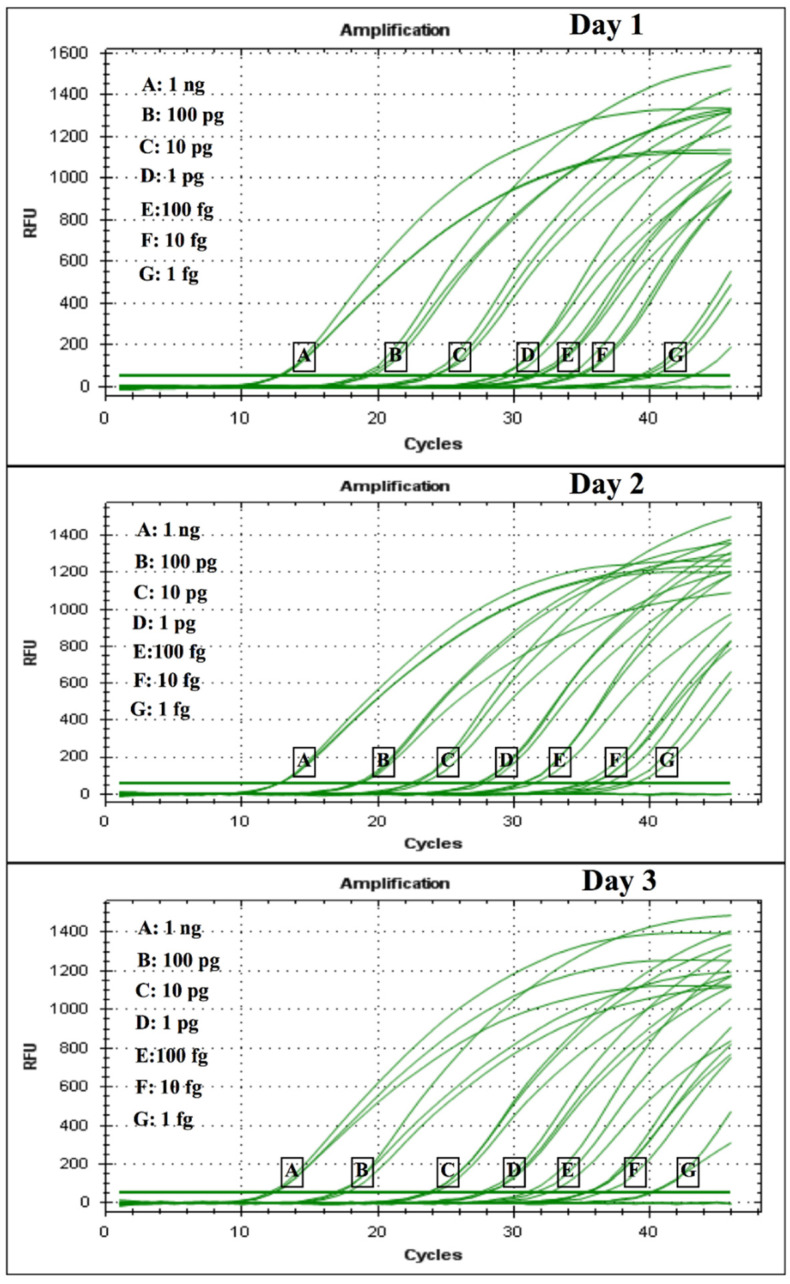
Standard curve for quantification obtained by using serial dilution 1:10 of different concentrations of DNA plasmid positive control (pCR 2.1 + PPK inset).

**Figure 2:**
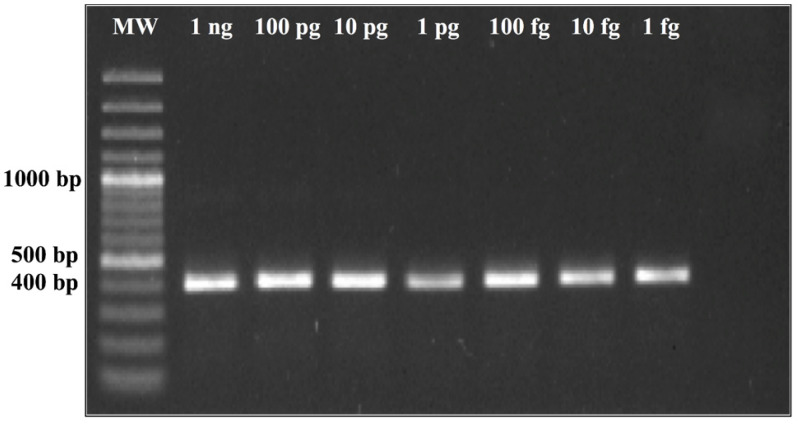
PPK control plasmid serial dilutions with nontemplate control (NTC).

In order to test for the analytical limit of detection (LOD) of the assays, 10-fold serial dilutions of positive control plasmids (pCR 2.1 + PPK insert) were performed from 1 ng down to 1 fg for both CFP4 and PPK plasmid controls. The dilutions were amplified via real-time PCR using standard conditions in triplicates for a sample processed on days 1, 2, and 3 for both plasmid controls (Supplemental Table S2). Detection of the positive control plasmid was observed up to 1 fg for all attempts for both *CFP4* and *PPK* plasmids. The LOD figure (Figure 1) shows very homogenous amplification curves for the *PPK*-positive control plasmid, while the LOD figure for the *CFP4* had higher variability within the day replicates. Although both regions showed low limits of detection, we recommend using the PPK primer pair based on the higher reproducibility observed. LOD was also tested via conventional PCR. A positive band was observed up to 1 fg for PPK (Figure 2).

### 3.3. Diagnostic Sensitivity in Stored FFPE Clinical Samples

**Figure 3:**
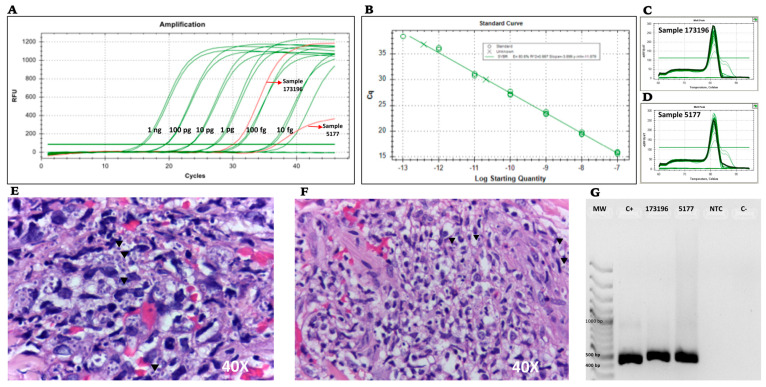
Detection of *H. capsulatum* DNA in FFPE skin tissue biopsies, qPCR, and melting curve analysis using PPK molecular target and histopathological stain. (**A**) qPCR amplification curves. (**B**) Standard curve. (**C**,**D**) Melting curve of PPK amplicons in clinical samples 173196 and 5177, respectively. (**E**,**F**) Microphotographs from skin biopsies stained with hematoxylin and eosin for samples 173196 and 5177, respectively. (**G**) Agarose gel electrophoresis of PPK conventional PCR amplification obtained from DNA extracted from FFPE skin biopsies. MW: molecular weight marker, C+: pCR2.1+PPK, NTC (nontemplate control), C-: human DNA.

The two FFPE clinical specimens we used for validation, which had been previously tested by fungal histopathology assay, showed amplification by conventional and real-time PCR using both primer sets. Conventional PCR detected an amplicon of approximately 400 bp for the PPK primer set, in line with expectations. In the qPCR, the CT values for samples 5177 and 173196 were 36.86 and 30.07, respectively. This could suggest that the fungal burden is lower, according to the number of yeast cells in the histological image and the CT values in Figure 3. Real-time PCR also showed that the melting curve for the PPK primer set generates a unique amplicon corresponding to the standard curve assayed in the run (Figure 3).

We obtained amplifications for both FFPE samples despite a lower fungal burden in the tissue stain for sample 5176 compared to sample 173196, confirming the high sensitivity of the assays designed using PPK primers for both PCR methodologies.

## 4. Discussion

We systematically designed and tested two primer pairs for detecting the fungal pathogen *Histoplasma* spp. that could be used for conventional or real-time PCR using similar conditions and the same primer sets. We focused on a general design to work for both PCR technologies, as specialized real-time PCR equipment may not always be accessible for molecular laboratories in resource-limited settings. The genomic regions in which the primer pairs were located correspond to two coding genes, culture filtrate protein 4 (*CFP4*) and a putative protein kinase, *PPK*. In view of its smaller PCR product size and more homogeneous amplification curves during LOD testing, we identified the PPK primer pair as the more promising choice for assay testing. The PPK primer pair was able to detect in vitro down to 1 fg of the control plasmid using both real-time and conventional PCR designs. The CFP4 primer pair gave good results, validating its analytic reliability and uniqueness to the *Histoplasma* genus. Although CFP4 was designed and considered, the large amplicon size did present some difficulties in consistent amplification, in the efficiency of real-time PCR curves and the linearity of the standard curves. In the practice, shorter amplicons with SYBR^®^ Green can record lower CTs than longer ones [23,24]. We observed in the CFP4 qPCR, which has a larger amplicon size (800 bp), higher CT values, low fluorescence intensity, and a very low efficiency (<50%). However, in the conventional PCR, the CFP4 amplicon showed more efficient amplifications in all strains tested. After these initial experiments, we decided to continue with the PPK primer design that, at 400 base pairs, did not show these difficulties during the PCR runs. 

We focused on the idea of creating primer sets that should work both for conventional and real-time PCR, and that do not involve a nested PCR design. Nested PCR requires two amplification steps, which can increase the complexity of the assay and the laboratory processing times and costs, as well as the probability of contamination. Assays using primer pairs obtained via our screening methods should not need a secondary amplification step, and in the designs we tested, the limits of detection were low enough to be comparable to a previous nested PCR assay of Bialek et al. in 2002 [11] targeting a region of a gene for a 100 kDa protein.

More recently, Lopez et al. [12] implemented real-time PCR assays for the detection of *H. capsulatum*, targeting Hc 100 kDa, H, and M antigens [11,12,13].

Our assay with PPK primers showed positive detections in 2 of 2 FFPE biopsies from patients with confirmed histoplasmosis. This finding suggests that the implementation of the PPK primer pair is likely to have good specificity for diagnosing histoplasmosis also in routine clinical settings.

In an environmental context, Gómez et al. [13] applied a protocol based on the amplification of the Hc100 nested PCR to search for *H. capsulatum* in composted organic fertilizers, soil samples from caves, and bird excreta, where only 10% of the samples were positive for the 100 kDa marker. 

The recently emerging fungal pathogen genus *Emergomyces*, which was named several years ago but has recently been more fully characterized, i.e., stably distinguished from its close relatives that are currently assigned to the genera *Emmonsia* and *Blastomyces*, is now represented by several genomic sequences, which also confirms its appreciable similarity to some other fungi from the Ajellomycetaceae family (see [25] and refs. therein). During our analysis, the only sequences that were available for *Blastomyces* were *B. dermatitidis*. Species such as *B. percursus* or *B. emzantsi* were not considered, due to the lack of sequences available. The latter should be considered for future analysis. In particular, this similarity has apparently led to noted problems in identifying *Emergomyces* in clinical settings where *Histoplasma* may also be present or vice versa, due to cross-reactions [25,26]. Specifically, our bioinformatic analysis shows that the 100 kDa primer sets have a 92.5% similarity with *Emergomyces* spp., where, e.g., in the genome sequence of *Emergomyces orientalis* (strain 5z489, scaffold 7), 37 out of 40 nucleotides corresponding to the forward and reverse primers have an exact match.

As the PCR assay for the detection of *H. capsulatum* amplifying the 100 kDa protein [11] is widely used for molecular diagnostics, we searched for the sequence of this gene in our OrthoMCL results. The results did not include this gene as a unique gene for *H. capsulatum*. For instance, BLAST results showed that the sequence for the 100 kDa gene, as well as the primers used in the assay, are similar to the closely related fungal species *B. dermatitidis* and *P. lutzii*. Success of the 100 kDa assay would therefore appear to be due to sufficient sequence dissimilarity in a gene that is, however, still present outside the *Histoplasma genus*.

At the time that we performed our experiments, the *Emergomyces* species had not been described. Moreover, we did not have genomic sequences or DNA available for in silico or in vitro analysis. The lack of these data can be considered a limitation in our analysis. When genomic reads became available for *Emergomyces* spp., additional in silico analysis was performed. We found that the primer sequences of PPK presented here have similarity to sequences in a region of *Emergomyces* spp., but with four mismatches in the forward primer and four mismatches in the reverse primer compared with *Histoplasma* spp., and, even in the event of an amplification, the amplicon length would be very different; in contrast to the amplicon length of 400 bp for *Histoplasma* spp., *Emergomyces* spp. would instead have a much larger amplicon of 750 bp. This indicates that our design for PPK primers would probably differentiate *Histoplasma* spp. from *Emergomyces* spp. using a basic PCR approach. 

The empirical results we present suggest that the putatively functional protein kinase gene used for the PPK assay may maintain evolutionary stability at the sequence level throughout strains of *H. capsulatum*, thus allowing consistent sensitivity of assays targeting the gene. Indeed, we confirmed in vitro the analytical sensitivity of the primer pair via testing of the DNA from 62 *H. capsulatum* strains of different clades representing a large geographical diversity, as described by Kasuga et al. [27], and we observed amplification in all 62 isolates tested. Further testing using two clinical samples positive for *H. capsulatum* that were available confirmed amplification in both cases; moreover, in one of the two FFPE samples, the fungal burden was low, yet amplification was also achieved in this sample. Although the results are promising, further testing in clinical samples is needed for confirmation of the clinical validity of the assay.

## 5. Conclusions and Perspectives

The *Histoplasma* PCR assays presented and partly validated in this study (in silico, analytically in vitro, and with FFPE skin samples of patients from a clinic) compare favorably with existing assays in their expected sensitivity and specificity in future clinical settings. In *Histoplasma*, however, a fair clinical performance evaluation of these and other assays might require independent verification of the fungus’ presence via another method in the same sample taken for the PCR assay, rather than just via illness of the patient or *Histoplasma* presence in a sample taken from a different tissue or at a different time. Such verification might be challenging if the life history of the infecting *Histoplasma* does not guarantee the fungus’ presence at all times in blood or tissues that can be easily sampled.

The choice of *Histoplasma* is a particularly stringent or ‘worst-case’ proof of principle of the assay design methodology presented here, in the sense that this fungus has close evolutionary and sequence similarity to other clinically important pathogens in the same family (Ajellomycetaceae), thus increasing the risk of cross-reactions/false positives unless special care is taken in choosing the primers. Thus, we consider that the lack of cross-reaction we observed across 25 other fungi in vitro is a nontrivial and reassuring result. 

Emerging fungal diseases of the past decade include emergomyces, in which new variants of previously existing strains from the *Blastomyces-Emmonsia* group evolved and rapidly spread; as a result, some previously specific PCR or antigen assays for detecting *Histoplasma* or other Ajellomycetaceae fungi may now have reduced discriminatory power in the clinic in countries where emergomyces has become endemic [25,26]. The approach we have presented here can frontally address the problem of a dynamically changing sequence landscape of pathogenic fungi, as it is based on empirical sequence comparison. Our design strategy would, in principle, allow the interleaving of assay updates with the arrival of new data streams from the molecular surveillance of endemic fungi (see, e.g., [28]). In the event that an existing assay design becomes less reliable following the appearance of new variants of concern, a list of alternative assay designs could be maintained and dynamically re-tested against newly emerging isolates’ sequences. For the ranking of assay candidates in such a list, notions and algorithms from abstract sequence informatics may be helpful, for example, those developed in the context of searching for or evaluating the longest distinguishing substrings ([29], Section 2.4 and Section 2.7). On a more general note, the need for agile assay designs that can be readily modified in response to emerging and clinically important species or strains (e.g., *Emergomyces* spp. or *Candida auris*) is also a priority need in other areas of microbiology, as the recent evolution of coronaviruses has made clear.

## Figures and Tables

**Table 1:** Non-*Histoplasma* species used for in vitro testing of analytical specificity.

Species	Isolate	Species	Isolate
*Coccidioides* *immitis*	CDC B6037, CDC B10637, CDC B10757, CDC B10813	*Candida* *tropicalis*	CIB Collection
*Blastomyces* *dermatitidis*	CDC B3591, CDC 26117, CDC 180017, CDC 26116, CDC 26114	*Candida* *parapsilopsis*	CIB Collection
*Aspergillus* *fumigatus*	CDC ATCC 1022 T	*Candida glabrata*	CIB Collection
*Aspergillus* *versicolor*	CDC NRRL238, CDC NRRL239	*Chrysosporium keratinophilum*	CDC B1959, CDC B1980, CDC B3644, CDC B2705
*Aspergillus flavus*	NRRL485, IFI 03-0139	*Cryptococcus* *neoformans*	B8915, B9029
*Aspergillus terreus*	CDC IBT14590, CDC 141	*Cryptococcus gattii*	B8558, B9300
*Aspergillus niger*	IFI03-0052, ATCC1015	*Uncinocarpus reesii*	CDC CBS 121.77
*Aspergillus* *fischeri*	B6256	*Pneumocystis jirovecii*	CDC 163
*Aspergillus pseudofischeri*	B5571, B5573	*Mycobacterium tuberculosis*	CIB Collection
*Paracoccidioides brasiliensis*	CIB Pb18, CIB Pb03	*Mycobacterium avium*	CIB Collection
*Paracoccidioides lutzii*	CIB Pb01	*Mycobacterium chelonae*	CIB Collection
*Candida albicans*	CIB Collection	*Mycobacterium fortuitum*	CIB Collection
*Meyerozyma* *guillermondi*	CIB Collection		

**Table 2:** Primer details.

Gene	Primer	Length (bp)	Temperature	GC%	Amplicon Size (bp)	Primer Sequence
*CFP4*	Forward	23	57.1 °C	52.2	800	5′-GTGACATCTGGAGCAGCTGTTGA-3′
	Reverse	23	57.1 °C	52.2		5′-TCAACTCGGGCGCTCTGTCAAAA-3′
*PPK*	Forward	22	54.8 °C	50	400	5′-CTGGTAAATAGGCGCTGTCTTG-3′
	Reverse	22	54.8 °C	50		5′-AGCTCAGCATCGACCGAATGAA-3′

## Data Availability

Sequence data used to derive or support reported results were retrieved from publicly available whole genome and nonredundant (nr) sequences archived in NCBI (genomes, nr) and/or the Broad Institute of MIT and Harvard (Fungal Genome Initiative), as specified with links in the main text of this article.

## Supplementary Materials

The following are available online at https://www.mdpi.com/article/10.3390/jof7070544/s1; Table S1. List of genomic sequences; Table S2. Standard curve CT values for PPK positive control plasmid LOD.
