# Supplementary material for: New Histoplasma Diagnostic Assays Designed via Whole Genome Comparisons
Source: J Fungi (Basel). 2021 Jul 9;7(7):544. doi: 10.3390/jof7070544 (PMC8305531; doi:10.3390/jof7070544)
Supplement: Supplementary file 1 [file jof-07-00544-s001.zip › jof-1242567-supplementary.pdf]

Supplemental material

Table S1. List of genomic sequences.

Name	Strain	Accession number	Sequencing technology	Genome coverage (X)
Sporothrix schenckii	ATCC58251	GCA_000474925.1	Illumina	56
Sporothrix schenckii	1099-18	GCF_000961545.1	454	17
Sporothrix schenckii	SsEM7	GCA_002837075.1	Illumina	128
Sporothrix schenckii	SsMS1	GCA_002941045.1	Illumina	127
Sporothrix brasiliensis	5110	GCA_000820605.1	454	20
Sporothrix globose	CBS 120340	GCA_001630435.1	Illumina	146
Sporothrix globose	SS01	GCA_001630445.1	Illumina	153
Grosmannia clavigera	kw1407	GCF_000143105.1	Sanger; 454; Illumina	64
Ophiostoma novo-ulmi subsp. novo-ulmi	H327	GCA_000317715.1	454	61
Ophiostoma piceae	UAMH 11346	GCA_000410735.1	Illumina; 454	735
Ophiostoma ips	CBS 138721	GCA_002917055.1	Illumina	54
Sporothrix insectorum	RCEF 264	GCA_001636815.1	Illumina	7037
Sporothrix pallida	SPA8	GCA_000710705.2	Illumina	50
Trichophyton benhamiae	CBS112371	GCF_000151125.1	454	7
Blastomyces dermatitidis	ATCC18188	GCA_000151595.1	454; ABI	25.52
Blastomyces dermatitidis	ER-3	GCA_000003525.2	ABI	9.4
Blastomyces gilchristii	SLH14081	GCA_000003855.2	ABI	8.4
Blastomyces percursus	EI222	GCA_001883805.1	Illumina	123
Coccidioides immitis	H538.4	GCA_000149815.1	ABI	3.41
Coccidioides immitis	RMSCC2394	GCA_000149895.1	ABI	8.22
Coccidioides immitis	RMSCC3703	GCA_000150085.1	ABI	3.17
Coccidioides immitis	RS	GCF_000149335.2	ABI	14.4
Coccidioides posadasii	RMSCC3488	GCA_000150055.1	ABI	8.52
Coccidioides posadasii	CPA0001	GCA_000150245.1	ABI	3.09
Coccidioides posadasii	CPA0020	GCA_000150615.1	ABI	3.42
Coccidioides posadasii	CPA0066	GCA_000150645.1	ABI	3.34
Coccidioides posadasii	RMSCC1037	GCA_000150555.1	ABI	3.41
Coccidioides posadasii	RMSCC1038	GCA_000150585.1	ABI	3.00
Coccidioides posadasii	RMSCC2133	GCA_000150185.1	ABI	6.69
Coccidioides posadasii	RMSCC3700	GCA_000150215.1	ABI	3.58
Coccidioides posadasii	Silveira	GCA_000170175.2	ABI	5

Emmonsia crescens	UAMH3008	GCA_001008285.1	Illumina	163
Emmonsia crescens	UAMH4076	GCA_002572855.1	Illumina	173
Blastomyces parva	UAMH130	GCA_002572885.1	Illumina	199
Blastomyces silverae	UAMH139	GCA_001014755.1	Illumina	116
Histoplasma capsulatum	G186AR	GCA_000150115.1	ABI	11.50
Histoplasma capsulatum	H143	GCA_000151035.1	ABI	3.82
Histoplasma duboisii	H88	GCA_000151005.2	ABI	7.77
Histoplasma capsulatum	Nam1	GCF_000149585.1	ABI	7
Histoplasma capsulatum	G217B	GCA_000170615.1	ABI	12.5
Microsporum canis	CBS113480	GCF_000151145.1	ABI	7.95
Nannizzia gypsea	CBS118893	GCF_000150975.2	ABI	9.06
Paracoccidioides americana	Pb03	GCA_000150475.2	ABI; Illumina	150
Paracoccidioides brasiliensis	Pb18	GCF_000150735.1	ABI; Illumina	198
Paracoccidioides restrepiensis	CNH	GCA_001713695.1	Illumina	129
Paracoccidioides venezuelensis	Pb300	GCA_001713645.1	Illumina	158
Paracoccidioides lutzii	Pb01	GCF_000150705.2	ABI; Illumina	148
Trichophyton equinum	CBS127.97	GCA_000151175.1	ABI	4.88
Trichophyton interdigitale	H6	GCA_000616785.1	Illumina	91
Trichophyton interdigitale	MR816	GCA_000622975.1	Illumina	56
Trichophyton rubrum	CBS100081	GCA_000616805.1	Illumina	73
Trichophyton rubrum	CBS202.88	GCA_000616985.1	Illumina	70
Trichophyton rubrum	CBS288.86	GCA_000616825.1	Illumina	167
Trichophyton soudanense	CBS452.61	GCA_000616865.1	Illumina	75
Trichophyton rubrum	CBS118892	GCF_000151425.1	ABI	8.19
Trichophyton rubrum	CBS289.86	GCA_000616845.1	Illumina	173
Trichophyton rubrum	CBS735.88	GCA_000616965.1	Illumina	55
Trichophyton rubrum	D6	GCA_000622995.1	Illumina	109
Trichophyton rubrum	MR1448	GCA_000616905.1	Illumina	174
Trichophyton rubrum	MR1459	GCA_000616945.1	Illumina	109
Trichophyton rubrum	MR850	GCA_000616765.1	Illumina	90
Trichophyton tonsurans	CBS112818	GCA_000616765.1	ABI	5.71
Trichophyton verrucosum	hki0517	GCF_000151505.1	ABI	3
Uncinocarpus reesii	1704	GCF_000003515.1	ABI	5
Emergomyces orientalis	5z489	GCA_002110485.1	Illumina; PacBio	410
Emergomyces pasteurianus	UAMH 9510	GCA_001883825.1	Illumina	88

Table S2. Standard curve CT values for PPK positive control plasmid LOD

Standard concentration	Day 1	Day 2	Day 3
**1 ng**	12,82 ± 0,12	12,62 ± 0,03	12,22 ±0,20
**100 pg**	19,11 ± 0,43	18,38 ± 0,20	17,20 ± 0,44
**10 pg**	23,93 ± 0,34	22,66 ± 0,43	23,82 ± 0,25
**1 pg**	29,31 ± 0,47	27,41 ± 0,31	27,83 ± 0,28
**100 fg**	31,60 ± 0,28	30,77 ± 0,52	31,71 ± 0,83
**10fg**	34,41 ± 0,50	35,34 ± 0,42	35,33 ± 0,08
**1fg**	39,74 ± 0,52	37,94 ± 1,05	38,94 ± 2,09
